# Multicenter, PrOspective, Randomized, Controlled Trial Comparing GenIcular Artery EmbOlization Using Embosphere Microspheres to Corticosteroid iNjections for the Treatment of Symptomatic Knee Osteoarthritis: MOTION Study Protocol Summary

**DOI:** 10.1007/s00270-025-03994-z

**Published:** 2025-03-06

**Authors:** Clare Bent, Craig J. McAsey, Sandeep Bagla

**Affiliations:** 1 Department of Interventional Radiology, Royal Bournemouth and Christchurch Hospital, Bournemouth, UK; 2 Anderson Orthopaedic Research Institute, Alexandria, VA USA; 3 Inova Mount Vernon Hospital Joint Replacement Center, Alexandria, VA USA; 4 Prostate Centers USA, LLC, Falls Church, VA USA

**Keywords:** Knee osteoarthritis, Genicular artery embolization, Embosphere® microspheres

## Abstract

**Purpose:**

Corticosteroid injections are commonly used to treat symptomatic knee osteoarthritis; however, pain relief is usually transient. Genicular artery embolization (GAE) has shown promise as an effective minimally invasive intervention to alleviate symptomatic knee osteoarthritis. The MOTION study is being conducted to compare outcomes following GAE versus corticosteroid injection.

**Materials and Methods:**

This is an international, multicenter, randomized controlled investigational device exemption (IDE) study enrolling adults (≥ 21 years old) with symptomatic knee osteoarthritis (Kellgren–Lawrence grades 1–4) across ≥ 45 centers worldwide. Patients will be randomized 1:1 to receive GAE with Embosphere® Microspheres (Merit Medical Systems, Inc.) or corticosteroid injections. The primary efficacy measure is clinical success at 6 months, defined as ≥ 50% improvement in the Western Ontario and McMaster Universities Osteoarthritis Index (WOMAC) Pain Subscale. The primary safety measure is the proportion of patients free from treatment-related safety event(s) through 6-month post-index procedure (GAE or corticosteroid injection). Following the 6-month visit, patients in either cohort who do not achieve clinical success may crossover to the other study arm. Additional study measures will assess safety and efficacy outcomes throughout the 24-month follow-up period. The target sample size is 264 (132 per arm) and is based on the number of patients needed to confirm superior efficacy of GAE versus corticosteroid injections and non-inferiority with respect to safety. The overall study power is > 80%.

**Discussion:**

Findings from the MOTION study are expected to provide information on the magnitude of the therapeutic benefits associated with GAE versus standard of care over 24 months.

**Trial Registration:**

NCT05818150.

**Graphical Abstract:**

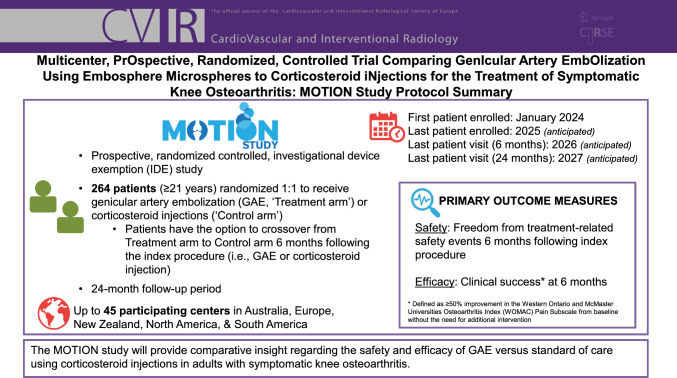

## Introduction

Knee osteoarthritis is a common degenerative condition that can result in debilitating pain. Recognized risk factors associated with the development of knee osteoarthritis include obesity, repetitive joint stress and trauma, advancing age, diabetes, and family history [1]. Over the last several decades, the incidence and prevalence of knee osteoarthritis have steadily increased. From 1990 to 2019, the prevalence of knee osteoarthritis has increased by 7.5% and the number of new cases has increased by 6.2% [2]. In 2020, 654.1 million adults ≥ 40 years of age were estimated to suffer from knee osteoarthritis globally [3]. As the risk factors associated with the development of knee osteoarthritis increase, the incidence and prevalence of the condition will continue to rise. The increasing clinical burden of knee osteoarthritis is also associated with a high economic burden throughout the world [4–6]. A retrospective claims-based study published in 2017 estimated that direct annual costs of knee osteoarthritis in the USA (e.g., treatment, medical visits) incurred by patients ranged from $5.7 to 15 billion [5].

Intra-articular corticosteroid injections are commonly used as the standard of care to alleviate pain associated with knee osteoarthritis; however, their use typically provides short-term relief (e.g., 4–6 weeks of benefit) [7]. Moreover, there is evidence to suggest that repeat corticosteroid injections may be associated with significant cartilage loss without meaningful long-term pain relief [8].

Within the past decade, genicular artery embolization (GAE) has emerged as a minimally invasive treatment helpful in reducing, or eliminating, the pain associated with knee osteoarthritis [9–15]. It has been hypothesized that “pruning” of the hypervascularity in the synovial tissues surrounding the knee joint can reduce inflammatory processes and provide pain relief. If confirmed, these have the potential to result in meaningful improvements in patients’ health-related quality-of-life [10, 12–14, 16–18].

Considerable evidence has demonstrated the clinical utility of GAE; however, most studies have focused on single patient cohorts or compared outcomes against sham procedures. The MOTION study has been designed to directly compare clinical outcomes of GAE versus standard of care intra-articular corticosteroid injections.

## Methods

### Study Design

The MOTION study is a prospective, multicenter, randomized, investigational device exemption study that is being conducted at up to 45 centers across Australia, Europe, New Zealand, North America, and South America. The study is sponsored by Merit Medical Systems, Inc. (South Jordan, UT, USA; Trial Registration: NCT05818150).

### Eligibility Criteria

Table 1 summarizes the full list of eligibility criteria. Patients with confirmed knee osteoarthritis who meet all of the general inclusion criteria and none of the general exclusion criteria will be randomized into one of two arms: the treatment arm (i.e., GAE) or the control arm (i.e., corticosteroid injection). Patients who are assigned to the treatment arm are screened to ensure that they meet all angiographic eligibility criteria (Table 1) before they can undergo GAE. Patients randomized to the treatment arm who do not meet the angiographic eligibility criteria are considered an angiographic screen failure and will be followed through discharge only. Any patient who is randomized into the study, but later found to have not met all eligibility criteria will remain in the study and complete all trial testing and follow-up requirements.

**Table 1:** Key inclusion and exclusion criteria

*General inclusion criteria*
Provides written informed consent Age ≥ 21 years Mild to severe knee pain, defined as a WOMAC Pain score of ≥ 8 out of 20 (in the target knee) Pain refractory to conservative therapies (e.g., anti-inflammatory drugs, or physical therapy, or muscle strengthening, or intra-articular injections) for at least 90 days prior to enrollment/randomization Kellgren–Lawrence grade 1, 2, 3, or 4 on radiograph of the knee within 6 months of enrollment/randomization (of the target knee)
*General exclusion criteria*
Planned major surgical or endovascular procedures ≤ 30 days after the index procedure Advanced atherosclerosis Known history of rheumatoid or infectious arthritis Received corticosteroid injection(s) in the target knee ≤ 90 days prior to treatment following enrollment/randomization Prior knee replacement surgery of the target knee
*Angiographic inclusion criteria*
Patient has confirmed evidence of knee osteoarthritis, defined as an angiographic ‘blush’ pattern (radiographic) in one or more of the target genicular artery(ies) Patient requires ≤ 4 genicular arteries to be treated
*Angiographic exclusion criteria*
Patient has evidence of arterial occlusion precluding catheterization (e.g., inability to cross iliac or superficial femoral artery(ies) and gain access to the genicular arteries) Patient has total occlusion of the genicular arteries on the target knee

WOMAC, The Western Ontario and McMaster Universities Arthritis Index

### Compliance with Ethical Standards

This study complies with the 1964 Helsinki Declaration and its later amendments. Ethics approval has been granted by the local ethics committees for each center participating in the study.

### Study Cohorts

Patients with symptomatic Kellgren–Lawrence Grades 1–4 (i.e., mild to severe) knee osteoarthritis will be randomized 1:1 into the treatment arm (GAE) or control arm (corticosteroid injection).

All randomized patients who do not achieve clinical success at 6 months will have the opportunity to crossover to the other study arm (i.e., crossover from active control to treatment [CtT], crossover from treatment to active control [CtC]). Crossover may only be selected following completion of the 6-month follow-up visit and quality-of-life assessment. Crossover will not be allowed at any subsequent time points during the study.

### Study Procedures

#### Screening

Any patient presenting to the participating centers with symptomatic knee osteoarthritis will be evaluated for eligibility and participation in the study (Table 1). If the patient meets the general eligibility criteria and is willing to participate in the study, written informed consent will be obtained. No study-specific requirements will be performed prior to obtaining informed consent from the patient.

#### Enrollment and Randomization

The MOTION study will enroll 264 patients across up to 45 centers, with a maximum of 15 centers allowed to be outside of the USA. Enrollment will not exceed 20% of total study population at any one center.

Patients will be randomized (1:1) to GAE or corticosteroid injection following confirmation that all eligibility criteria are met (Table 1).

#### Intervention

##### Treatment Arm

For patients randomized to the treatment arm (GAE), a baseline angiogram will be obtained to determine final eligibility prior to inserting the embolic agent into the vasculature.

**Fig. 1:**
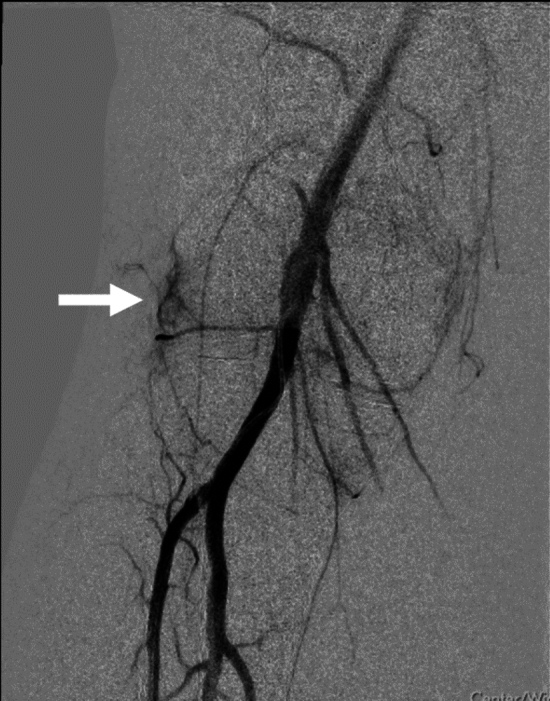
Genicular artery angiogram demonstrating “contrast blush” denoting hypervascularity (white arrow)

An intra-arterial sheath will be placed via femoral access (ipsilateral or contralateral) for the GAE procedure. Radial and pedal access are not permitted in this trial. Prior to treatment with the embolic agent (Embosphere® Microspheres, 100–300 µm; Merit Medical Systems, Inc.), angiography will be performed according to standard of care at each participating center and in accordance with requirements from the core laboratory, to confirm the presence of hyperemia (i.e., ‘blush’ pattern, Fig. 1) within the synovial tissues surrounding the affected knee joint in accordance with the angiographic criteria (Table 1). If confirmed, the genicular artery(ies) will be assessed to identify culprit vessels contributing to the hypervascularity of the synovial tissue in the region of the knee joint.

Under fluoroscopic guidance, the embolic agent will be injected with attention to avoid reflux and non-target embolization until adequate pruning of the hypervascularity is achieved (i.e., cessation of blood flow). Embolization will target small caliber distal vessels while maintaining patency of the larger main genicular artery. A maximum of four target vessels may be treated.

A final lower extremity angiogram will be performed to confirm success of the embolization procedure and determine if there are any associated complications. The patient will be discharged in accordance with each institution’s standard of care. Patients may be prescribed pain medications to treat post-procedure pain (e.g., non-steroidal anti-inflammatory drugs and/or other analgesics) according to the physician’s discretion. Medications prescribed to the patient will be documented in the case report form.

Patients who undergo GAE cannot receive concomitant treatment with a corticosteroid injection to manage procedural pain and inflammation.

##### Control Arm

For patients randomized to the control arm, the corticosteroid administered will be selected based on the standard practice and availability within the region of practice and limited to 40 mg of either triamcinolone or methylprednisolone. Both are long-acting steroids commonly used in the knee and a 40 mg dose has demonstrated activity out to 24 weeks [19, 20]. Only local topical or subcutaneous anesthetic may be used to manage local discomfort associated with the injection site procedure. Intra-articular steroid injections are not recommended more frequently than every 6 months, and patients are strongly discouraged from repeat injections at intervals less than every 6 months. Reintervention, including repeat injection, prior to the 6-month primary endpoint is considered a treatment failure per protocol.

The steroid will be injected into the joint cavity according to the physician’s discretion utilizing aspiration technique of synovial fluid or via ultrasound imaging to confirm presence within the joint cavity.

### Primary Outcome Measures

The primary efficacy outcome measure is the proportion of patients achieving clinical success at 6 months (180 days), defined as ≥ 50% improvement in the Western Ontario and McMaster Universities Osteoarthritis Index (WOMAC) Pain Subscale from baseline without the need for additional intervention (i.e., rescue treatment for inadequate pain relief such as intra-articular steroid injection or other interventions, exclusive of vascular intervention such as GAE).

The primary safety outcome is the freedom from treatment-related safety events through 6 months (180 days) following the index procedure.

### Additional Outcome Measures

Secondary outcome measures will include: the number of patients who achieve clinical success at 3, 12, and 24 months; patient rating of pain at baseline, 3, 6, 12, and 24 months as assessed using the Numerical Rating Scale (NRS); the number of patients who achieve ≥ 50% reduction in the mean NRS pain score at 3, 6, 12, and 24 months. Additional secondary outcome measures will include assessments of technical success (defined as successful procedural occlusion of the target genicular arteries confirmed fluoroscopically by the treating physician), device-related and serious adverse device effects through 24 months, in addition to symptom-related changes throughout the study period. Exploratory endpoints will include, as available, the number of patients that progress to partial or total knee replacement and outcomes in any crossover patients will also be described.

### Follow-Up

During the 24-month study period, data will be collected at the following post-treatment/procedure timepoints: 3 days, 1, 3, 6, 12, and 24 months (Table 2). A standardized electronic case report form will be used to collect clinical data across all participating centers. As available, collected data will include demographics, medical history, physical assessments, medications, magnetic resonance imaging, quality-of-life questionnaires (e.g., 12-Item Short Form Health Survey, WOMAC), pain assessment via completion of the NRS, and adverse events. Data will be entered into the secure, web-based, electronic case report form. The inputted data will be reviewed by a combination of automatic programmed queries and manual review to identify inconsistent and missing data entries for resolution.

**Table 2:** Data collection schedule

Data	Baseline^a,b^	Day 0 Index procedure	3 days (3 ± 2 days) (Phone)	1 month (30 ± 7 days)	3 months (90 days ± 10 days)	6 months (180 ± 15 days)	12 months (360 ± 30 days)	24 months (720 ± 60 days)
Informed consent^c^	X							
Inclusion/exclusion criteria evaluation	X	X				X^d^		
Demographics	X							
Medical history	X							
Physical examination	X			X	X	X	X	X
Pregnancy test (if applicable per standard of care)^b^	X					X^d^		
Medications (NSAIDs/corticosteroids/ analgesics)	X	X		X	X	X	X	X
MRI	X^e^					X^e,f^	X^f^	
WOMAC	X			X	X	X	X	X
SF-12	X			X	X	X	X	X
Numerical Rating Scale	X			X	X	X	X	X
Adverse events^g^	X^g^	X	X	X	X	X	X	X

MRI, magnetic resonance imaging; NSAIDs, non-steroidal anti-inflammatory drugs; SF-12, 12-Item Short Form Health Survey; WOMAC, The Western Ontario and McMaster Universities Arthritis Index

^a^Standard of care assessments may be done up to 30 days before the procedure. Protocol-specific assessments/examinations that are non-standard of care cannot be obtained until after informed consent is obtained

^b^Pregnancy test (sites should follow their institutional policy/standard of care for pregnancy testing in females of child-bearing age prior to angiography)

^c^Informed consent to be obtained within 30 days prior to enrollment

^d^Crossover patients only

^e^Baseline MRI required of both groups—may be obtained up to 180 days prior to randomization/enrollment; 6-month follow-up MRI required of treatment group only (GAE group)

^f^MRI at 6-month visit and 12-month visit only required for patients that crossover to GAE treatment (not applicable to GAE to steroid injection crossover)

^g^Adverse events to be collected beginning at time of treatment following randomization

Any patient who receives a partial or total knee replacement of the targeted knee, or who is non-compliant will be withdrawn. Investigators may also withdraw any patient from the study that is deemed to be in the best interest of the patient. Patients will be encouraged to complete a final study exit visit at the time of withdrawal to assess for safety. Any data collected up to the time the patient is withdrawn from the study will be maintained as part of the study record and analyzed as appropriate.

If a patient discontinues the study at any time, is withdrawn from the study early, or completes all protocol required follow-up, they will be followed per the local standard of care for their condition.

Any patient who withdraws from the trial, or is lost to follow-up, will not be replaced.

## Data Monitoring

Throughout the study, the sponsor will conduct monitoring visits using a risk-based approach to verify that the study is being conducted in accordance with the protocol and good clinical practice, and to verify that critical data points, endpoint and safety data are accurately and completely documented in accordance with source medical records. The study will be audited in accordance with the sponsor’s audit program, including audit of investigational sites.

Safety events will be classified according to the International Organization for Standardization (ISO 14155:2020) definitions. An independent Clinical Events Committee (Yale Cardiovascular Research Group), of at least three independent physicians with experience performing GAE, will be responsible for systematic review and adjudication of specified safety events.

### Statistical Analysis

The proportion of the patients in the treatment and control arms achieving clinical success at 6 months will be compared. The hypothesis that treatment (GAE) is superior to control (corticosteroid injection). The number of patients needed to assess clinical success at 6 months was determined using a two-group Chi-square test based on the following assumptions: a clinical success rate of 65% for the treatment arm [12, 16] and a 40% clinical success rate for the control arm [8, 20, 21].

For the safety endpoint, treatment is hypothesized to be noninferior to control. The number of patients needed to assess the primary safety outcome was based on the following assumptions: (1) event-free safety rate of 95% in the treatment arm [11–14, 16, 22–24]; (2) event-free safety rate of 95% in the control arm [8]; (3) non-inferiority margin of 10%; (4) one-sided alpha of 0.025; (5) 85% statistical power; and (6) 15% attrition rate.

A required sample size of 264 patients (132 per arm) inclusive of a 15% attrition was determined. The overall study power is > 80%.

For purposes of endpoint determinations, the intent-to-treat (ITT) analysis set will include all randomized patients in both arms, and the modified ITT (mITT) analysis set will include all ITT patients who receive treatment and exclude patients that failed angiographic screening. The mITT analysis set will be used to evaluate the primary efficacy and safety outcome measures.

Continuous study measures will be summarized using descriptive statistics (mean, standard deviation, median, minimum, and maximum). Categorical or binary outcomes will be summarized using percentage with confidence intervals as appropriate.

Kaplan–Meier plots will be used to summarize time-to-event data. Continuous variables will be evaluated with independent t-tests. The threshold for statistical significance is *p* < 0.05.

Outcomes of crossover patients will be reported on a mITT basis and analyzed as separate populations (CtT and CtC).

### Expected Gain of Knowledge

Most available evidence has consistently demonstrated the clinical utility of GAE for patients with symptomatic knee osteoarthritis. Initial reports of clinical outcomes following GAE focused on the off-label use of the antibiotic imipenem/cilastatin, a non-permanent embolic [12, 13]. Although subsequent evidence has evaluated the safety and effectiveness of GAE following use of permanent embolic agents over the longer term, each of these studies were conducted in a small sample of patients treated at a single center [11, 14, 16]. A recent double-blind randomized control trial describing clinical outcomes of GAE versus a sham-control procedure reported no significant difference in the clinical effect of GAE [25]. However, this trial was conducted at a single center and included 58 patients total, 29 treated with GAE and 29 randomized to the sham procedure [25].

In recognition of the need for ongoing evidence confirming the clinical benefits of GAE in larger patient cohorts across multiple centers, there has been an increase in the number of trials [9] underway to address this knowledge gap with one study comparing outcomes of permanent (Embosphere) versus temporary (imipenem/cilastatin) embolic agents [26]. An important evidence gap that the MOTION study will fill is how GAE with a permanent embolic compares to standard of care corticosteroid injections. This is an important aspect for clinicians who are hesitant to consider GAE without comparative evidence regarding the efficacy and safety of this intervention versus standard of care. While the primary outcome measures will focus on results at 6 months, data will be collected over a 24-month period, which is widely recognized as a key timepoint reflective of medium to longer term follow-up, which may help broaden our understanding regarding outcomes in the longer term. Moreover, the MOTION study will provide information regarding the proportion of patients who crossover, allowing a more complete understanding regarding the durability of clinical benefits. Currently, 38 of 45 sites have been selected.

## Data Availability

Not applicable.

## Declarations

### Human and Animal Participants

All procedures performed in studies involving human participants were in accordance with the ethical standards of the institutional and/or national research committee and with the 1964 Helsinki Declaration and its later amendments or comparable ethical standards.
